# Origin and function of the yolk sac in primate embryogenesis

**DOI:** 10.1038/s41467-020-17575-w

**Published:** 2020-07-28

**Authors:** Connor Ross, Thorsten E. Boroviak

**Affiliations:** 1 Department of Physiology, Development and Neuroscience, University of Cambridge, Downing Street, Cambridge, CB2 4BG UK; 2 Wellcome Trust-Medical Research Council Cambridge Stem Cell Institute, University of Cambridge, Jeffrey Cheah Biomedical Centre, Puddicombe Way, Cambridge Biomedical Campus, Cambridge, CB2 0AW UK; 3 Centre for Trophoblast Research, University of Cambridge, Downing Street, Cambridge, CB2 4BG UK

**Keywords:** Developmental biology, Reproductive biology

## Abstract

Human embryogenesis is hallmarked by two phases of yolk sac development. The primate hypoblast gives rise to a transient primary yolk sac, which is rapidly superseded by a secondary yolk sac during gastrulation. Moreover, primate embryos form extraembryonic mesoderm prior to gastrulation, in contrast to mouse. The function of the primary yolk sac and the origin of extraembryonic mesoderm remain unclear. Here, we hypothesise that the hypoblast-derived primary yolk sac serves as a source for early extraembryonic mesoderm, which is supplemented with mesoderm from the gastrulating embryo. We discuss the intricate relationship between the yolk sac and the primate embryo and highlight the pivotal role of the yolk sac as a multifunctional hub for haematopoiesis, germ cell development and nutritional supply.

## Abstract (web summary)

Why do human embryos need a yolk sac and how does it form? This Perspective by Thorsten Boroviak and Connor Ross explores the development and function of the yolk sac in primate embryogenesis.

## Introduction

The yolk sac is phylogenetically the oldest extraembryonic membrane to support embryogenesis. It evolved in our aquatic ancestors >500 million years ago^1^ and its original function was to absorb nutrients deposited in the yolk. Despite the absence of yolk in the eggs of placental mammals, the yolk sac has remained an integral part of embryonic development. Human and non-human primates are no exception.

The mammalian yolk sac originates from the hypoblast (also known as primitive endoderm), which is specified at the late blastocyst stage prior to embryo implantation. In human and non-human primates, yolk sac formation entails the generation of a transient primary yolk sac—before the establishment of a secondary yolk sac. Primary yolk sac formation coincides with the emergence of extraembryonic mesoderm after implantation. Extraembryonic mesoderm is an integral part of amnion, yolk sac, allantois and chorion (Box 1), with critical roles in vascularisation and nutrient transport. Rodent extraembryonic mesoderm originates from the *embryo proper* during gastrulation. Interestingly, primate extraembryonic mesoderm is specified prior to gastrulation and the developmental origin of this early mesoderm population has been subject to intensive debate.

In this review, we briefly touch upon the evolutionary origin of the yolk sac and subsequently shift our focus towards the developmental origin of the primate yolk sac. We collate our current knowledge on the emergence of the yolk sac’s founding population, the hypoblast, its progression to the primary yolk sac in the implanting primate embryo and the establishment of the secondary yolk sac. Moreover, we propose a rationale for primary yolk sac formation and early extraembryonic mesoderm specification in primates. We discuss the critical roles of the secondary yolk sac for haematopoiesis, germ cell development and nutritional transport during early primate embryogenesis and conclude by providing an outlook on the exciting prospect of deriving human and non-human primate hypoblast cultures to model yolk sac development in vitro.

Box 1 Extraembryonic membranes in human development Amnion: The innermost extraembryonic membrane consisting of a fluid-filled sac surrounding the embryo. Amnion provides a protective aqueous environment for the developing foetus.

Yolk sac: Human yolk sac development consists of two developmental phases: Initially, the embryo forms a primary yolk sac, which rapidly collapses and is replaced by a secondary yolk sac. The secondary yolk sac is the definitive yolk sac. It gives rise to the first blood cells of the embryo and is highly vascularised. The secondary yolk sac ensures nutritional supply for the early embryo before the chorion is sufficiently developed to perform this function.

Allantois: An extraembryonic membrane that extends from the yolk sac into the connecting stalk. Together with the yolk sac, it acts as a source for embryonic blood cells.

Chorion: The outermost extraembryonic membrane derived from the trophoblast of the blastocyst. Together with the allantois, the chorion represents the foetal component of the placenta and is highly vascularised for gas exchange, waste management and nutrient transport during foetal growth.

## Evolutionary origin of the primate yolk sac

**Fig. 1:**
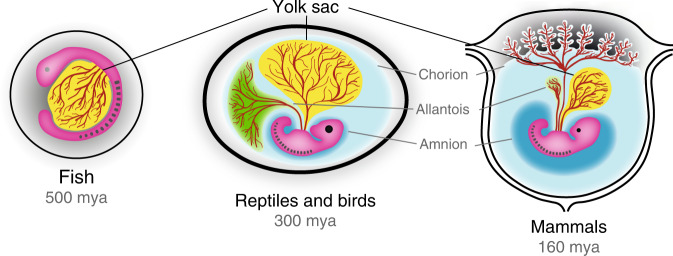
Extraembryonic membranes in fish, birds and mammals. The yolk sac dates back to our aquatic ancestors and thus represents the phylogenetically oldest extraembryonic tissue. Amnion, chorion and allantois are inventions of the amniotic egg, which have been subsequently adapted in mammals to support embryonic development inside the uterus. mya million years ago.

Successful reproduction on land required substantial rearrangements of the aquatic egg, in particular with regard to gas exchange, waste disposal and protection from desiccation^2,3^. To accomplish this challenge, amniotes (the clade of reptiles, birds and mammals) have developed three additional extraembryonic membranes: amnion, chorion, and allantois^3^ (Fig. 1). The evolution of amniotic eggs with large yolk-filled sacs, extensive extraembryonic tissues and less gelatinous shells effectively liberated amniotes from aquatic environments and allowed them to conquer dryer habitats inland. Strikingly, all extraembryonic membranes of the amniotic egg, including the yolk sac, are conserved in viviparous (producing living young) eutherian mammals.

Viviparity has evolved independently many times across the animal kingdom^4^. The evolutionary advantages of viviparity include protection from predators and pathogens, as well as effective thermoregulation throughout the seasons^3,5^. In addition, nutritional resources can be delivered to the offspring in smaller quantities, rather than all at once, and viviparous parents are freed from the demands of tendering immobile eggs^4^. Although this mode of reproduction impacts litter size due to limited available space, viviparity has proven evolutionarily advantageous to higher vertebrates with longer gestation times.

In most cases, viviparity gradually developed by retention of the egg in the mother for prolonged periods of time before hatching until the point at which the egg is kept entirely internal and the newborn baby emerges. Non-mammalian vertebrates and one of the four egg-laying mammals, the platypus, rely on nourishment from yolk rich in vitellogenin during the gestation period. Genomic comparison with chicken vitellogenin showed conserved sequences in human, armadillo and dog; however, the vitellogenin homologues in viviparous species have been degenerated by frame-shift mutations and premature stop codons^6^. In contrast, the platypus genome contains two *VIT* genes (*VIT1 and VIT2)*, where *VIT1* is eroded but *VIT2* remains functional for an oviparous mode of reproduction. Major milk resource genes, such as caseins, appeared before the loss of *VIT* genes in the common mammalian ancestor^6^. This suggests that the emergence of lactation and placentation allowed for the gradual loss of yolk-dependent nourishment during mammalian evolution^6^.

An important consideration is that mammalian viviparity emerged comparatively late and had to be superimposed on the existing structures of the amniotic egg^4^. Thus successful adaptation for in utero development required repurposing of extraembryonic membranes (Fig. 1). As in reptiles and birds, mammalian extraembryonic membranes function as surrogate lung, gut, liver and kidney, long before these organs are formed in the foetus^7^. Mammals establish a cooperative network of extraembryonic tissues consisting of amnion, chorion, yolk sac and allantois (Box 1). The amnion is a transparent membrane, which forms a fluid-filled sac surrounding the embryo. It provides an aquatic environment for the conceptus to prevent desiccation, ensures free movement of the foetus and serves as a shock absorber throughout gestation. While the amnion is an avascular membrane in most mammals, the yolk sac, allantois and chorion are highly vascularised to maximise nutrient and gas exchange between mother and foetus. The yolk sac functions as an absorptive epithelium for nutrient uptake and secretion as well as the origin of the first blood cells. In human and non-human primates, the allantois is a small diverticulum, which is part of the umbilical cord, connects to the bladder and acts as a temporary store for foetal excretions. The chorion is formed from extraembryonic mesoderm and trophoblast. Depending on the species and developmental stage, the chorion fuses with either the yolk sac to form the choriovitelline placenta or the allantois to form the chorioallantoic placenta. In most mammals, the choriovitelline placenta supports the early stages of development, while later on, the definitive, chorioallantoic placenta takes over to ensure sufficient nutritional supply during the foetal growth phase. Notably, there are numerous exceptions, including marsupials, which predominantly feature choriovitelline placentas, and rodents, which maintain both placental types throughout gestation^8,9^.

The yolk sac is among the first extraembryonic membranes to develop. In human and non-human primates, the yolk sac never physically attaches to the chorion in the chorioallantoic placenta^10^. Instead, it is linked to the embryo via the vitelline duct and floats freely in the exocoelomic cavity. After the first trimester, when the uteroplacental circulation is established as the main source for oxygen and metabolite exchange, the yolk sac degenerates and is often absent at birth^11^. For this reason, the yolk sac was once considered a mere remnant of our evolutionary ancestry. However, in recent years there has been accumulating evidence suggesting that the yolk sac is the main nutritional supply line to the embryo prior to the establishment of the intervillous circulation of the placenta^12–16^. To understand how the yolk sac accomplishes its vital functions, we have to go back to its origin in the preimplantation embryo.

## Development of the yolk sac

### Establishing the founding population: hypoblast specification in the primate blastocyst

The mammalian yolk sac is derived from the hypoblast, an extraembryonic lineage originating from the early inner cell mass (ICM) of the blastocyst. Rodent models have provided invaluable mechanistic insights into hypoblast formation (reviewed in refs. ^17–22^). However, recent studies highlight considerable differences in transcriptional regulation and signalling pathway deployment in human and non-human primate hypoblast specification^23,24^.

At a first glance, primate preimplantation development appears to follow the rodent paradigm, albeit developmentally protracted. Embryonic genome activation occurs at the eight-cell stage^25,26^, instead of the two-cell stage in mouse^27^. Primate embryos initiate compaction around the 16–32-cell stage, one cell division later than the mouse and form an early blastocyst by the 64-cell stage. Compaction of the morula inaugurates the first lineage decision, when outer cells give rise to trophoblast and interior cells become the ICM of the early blastocyst. A distinctive feature of early ICM cells in rodents is co-expression of the hypoblast specifier *Gata6* and the pluripotent epiblast marker *Nanog*^28^. The transcriptomes of individual ICM cells are indistinguishable at the 32-cell stage^29^; however, this homogeneity is short lived. Within hours, small transcriptional changes progressively manifest^29,30^ and NANOG and GATA6 protein expression becomes mutually exclusive, resulting in a “salt and pepper” pattern at the 64-cell stage^28,31^. The transcriptional programme for mouse hypoblast specification involves the sequential upregulation of *Gata6*, *Sox17*, *Gata4* and *Sox7*^32^.

In accordance with the mouse model, primate lineage specifiers *NANOG* and *GATA6* are initially co-expressed in the early ICM^24,33,34^ and resolve into discrete epiblast and hypoblast populations prior to implantation^24,33–35^. Comparative transcriptome analysis of human and non-human primate preimplantation stages showed that sequential activation of *GATA6*, *SOX17* and *GATA4* is conserved; however, *SOX7* is absent^23,24,34,36^. Early primate hypoblast markers—expressed in early ICM and sustained in hypoblast but downregulated in epiblast—include *GATA6*, *PDGFRA*, *TBX3* and *HNF4A*^34,36^, while *GATA4*, *SOX17*, *HNF1B*, *APOA1* and *OTX2* constitute late markers, upregulated in the mature primate hypoblast of the late blastocyst^34,36^.

Mouse hypoblast formation is critically dependent on fibroblast growth factor (FGF) signalling, as the emerging hypoblast population sustains *Gata6* expression in response to FGF4 secreted by *Nanog*-positive epiblast progenitors^17,37–39^. *Gata6* is functionally required for hypoblast specification^40–42^, but its initial expression is independent of *Fgf4*^43,44^. Transcriptome analysis showed that FGF receptors *Fgfr2*, *Fgfr3* and *Fgfr4* are hypoblast specific, while *Fgfr1* is expressed in all cells^24,29^. However, both *Fgfr1* and *Fgfr2* mediate FGF signalling during hypoblast development and loss of both receptors completely abolishes hypoblast formation^45^. In line with the genetic evidence, pharmacological inhibition of Mek/Erk signalling eliminates hypoblast formation and promotes epiblast^46^, while activation of FGF signalling favours hypoblast development at the expense of epiblast specification^47^. Collectively, this demonstrates a dominant role of FGF signalling in mouse hypoblast lineage acquisition.

Primate early ICM cells exhibit increased expression of WNT, transforming growth factor β (TGFβ)/NODAL and bone morphogenetic protein (BMP) pathway components^23,24,34^. *RSPO3*, a potent WNT signalling enhancer^48^, is one of the predominant primate-specific hypoblast markers^23,24,36,49,50^. Conversely, marmoset, cynomolgus monkey and human epiblast cells express WNT ligands, in contrast to the mouse^24,34,36^. *NODAL*, *TGFBR1*, *TGFBR3* and *ACVR1B* transcription is earlier and stronger than in the mouse, while *FGF4* is delayed in primates^24,36^. This divergence in signalling pathway component expression provides a rationale for the fact that FGF signalling inhibition does not entirely block hypoblast formation in human^33,51^. Comparative embryo inhibitor experiments in marmoset and mouse showed that blocking of either FGF or WNT signalling slightly reduced hypoblast cell numbers, while combined inhibition ablated hypoblast specification^24^. This suggests that primate hypoblast specification is regulated by multiple pathways, including FGF and WNT, in contrast to mouse, where FGF signalling is the predominant driver for lineage segregation.

By the late blastocyst stage, the rodent ICM has resolved into mature epiblast and hypoblast lineages as a result of cell migration and apoptosis^31^. Mouse ICM cells progressively lose developmental plasticity and become irreversibly committed to either lineage^52,53^. The hypoblast cells adjacent to the blastocyst cavity polarise and form an epithelium. This process is assisted by atypical protein kinase C (*Prkci*, *Prkcz*), which is required for acquisition of apical–basal polarity and, as such, hypoblast maturation^54^. Although much less is known about the molecular and functional characteristics of the late primate hypoblast, electron micrographs of rhesus embryos show that hypoblast cells equally consists of a continuous, polarised epithelium^55^. Therefore, epithelialisation constitutes the final step of hypoblast specification in both rodent and primate blastocysts, which are now ready for implantation.

### Primary yolk sac formation at the periimplantation stage

Embryo implantation is a developmental milestone, where the embryo undergoes major reorganisation. Extraembryonic tissues grow rapidly to establish a permanent link to the mother and secure a steady supply of metabolites and oxygen.

**Fig. 2:**
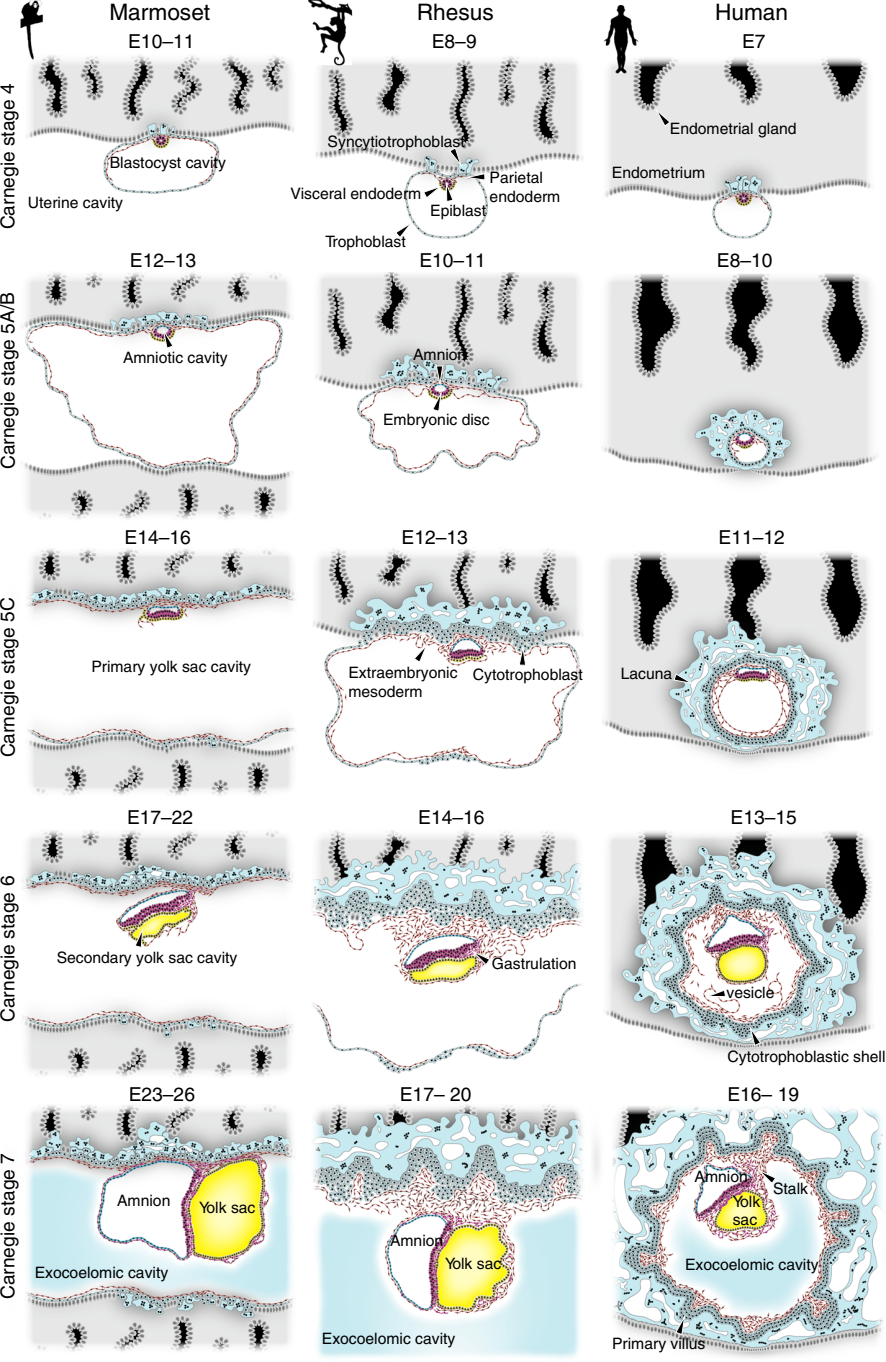
Yolk sac formation in primates. Embryonic stages of common marmoset (*Callithrix jacchus*), rhesus monkey (*Macaca mulatta*) and human (*Homo sapiens*) are depicted in Carnegie stages and embryonic day (E) for each species. The definitive, secondary yolk sac is highlighted in yellow. Drawings are based on representative histological sections of common marmoset^82,83^, rhesus^81^ and human^71,72,87,161,162^ embryos.

In great apes (human, chimpanzee, gorilla and orangutan) and lesser apes (gibbon)^56^, embryos undergo interstitial implantation, as the late blastocyst penetrates through the epithelial lining of the endometrium and invades into the underlying connective tissue. Embryos of other primate species, including Old World (rhesus and cynomolgus) and New World monkeys (marmoset), implant superficially, with the blastocyst expanding in the central cavity of the uterus^57^. Despite this difference in implantation mode, yolk sac formation consists of two developmental phases in probably all primates. In particular, human embryos develop a primary yolk sac between Carnegie stages (CS) 4 and 5, which is then replaced by a secondary yolk sac (Fig. 2). The secondary yolk sac is the definitive yolk sac and supports the conceptus during the first 2 months of pregnancy.

Primary yolk sac formation begins at the periimplantation stage at CS 4 (Box 2), when cells of the outer trophoblast layer of the blastocyst fuse to form syncytiotrophoblast and attach to the endometrium (Fig. 2). The hypoblast forms a squamous epithelium covering the epiblast^58^ and expands beyond the epiblast margin. At this stage, hypoblast cells diversify into visceral and parietal endoderm (Fig. 2). Visceral endoderm overlies the epiblast and gives rise to a cuboidal epithelium. The peripheral hypoblast cells become parietal endoderm, which forms an inner lining of the trophoblast. Both visceral and parietal endoderm contribute to the primary yolk sac^55,59^. The epiblast polarises into a rosette and undergoes cavitation, thereby segregating into amnion and embryonic disc. At CS 5, syncytiotrophoblast penetrates deeper into the endometrium and gives rise to small cavities, termed lacunae. In the meantime, parietal endoderm further expands and lines the inner cavity of the trophoblast. This process completes primary yolk sac formation by the end of CS 5.

Box 2 Chronology of human embryogenesis in Carnegie stages Developmental timing is indicated in embryonic days (E).

Carnegie stages (CS) in human:

CS 1 (E1): Fertilisation resulting in the formation of a unicellular embryo, the zygote.

CS 2 (E2–4): Cleavage divisions leading to morula formation.

CS 3 (E5–6): Blastocyst development and hatching from zona pellucida.

CS 4 (E7): Apposition and initial attachment of the blastocyst to the endometrium. Polarisation of the pluripotent epiblast.

CS 5A (E8): Epiblast segregates into amnion and the pluripotent embryonic disc. Syncytiotrophoblast formation and invasion of the endometrium.

CS 5B (E9–10): Expansion of the amniotic cavity. In the syncytiotrophoblast, small gaps appear, termed lacunae.

CS 5C (E11–12): Continuous lacunae formation leads to a nearly complete sphere; extraembryonic mesoderm begins to intrude into prospective primary chorionic villi.

CS 6A (E13): Initiation of gastrulation in the embryonic disc and primary chorionic villi formation.

CS 6B (E14–15): Primitive streak becomes clearly visible.

CS 7 (E16–19): Formation of the notochord and initiation of haematopoiesis in the yolk sac.

### Anterior visceral endoderm is essential for embryo patterning

In mammals, the anterior–posterior body axis is established through gradients of conserved signalling pathways (reviewed in refs. ^39,60,61^). Visceral endoderm plays a critical role in this process, as part of it forms a dynamic signalling centre, the anterior visceral endoderm (AVE). The AVE is specified prior to the formation of the primitive streak and essential for patterning of anterior regions in the mammalian conceptus^39,60–64^.

**Fig. 3:**
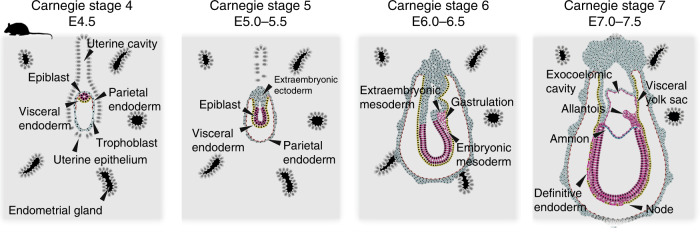
Early postimplantation development in mouse. Embryonic stages are depicted according to Carnegie stages and embryonic day (E)^39,60,61,163^.

Most of our knowledge about mammalian gastrulation is derived from studies in mouse (Fig. 3), where BMP4 signalling from the extraembryonic ectoderm is pivotal for setting up the proximal–distal axis^65^ and required for the generation of PGCs^66^. Extraembryonic ectoderm expresses proteases SPC1 (Furin) and SPC4 (Pace4), which activate NODAL in the epiblast^67^. At the border between epiblast and extraembryonic ectoderm, NODAL re-enforces BMP4 expression, which induces WNT3 in the proximal epiblast^68^. At the same time, NODAL expression from the epiblast specifies the most distal visceral endoderm cells to secrete NODAL inhibitors CER1 and LEFTY1, as well as WNT inhibitors DKK1, SFRP1 and SFRP5^39,61,69^. Distal visceral endoderm cells subsequently migrate towards the prospective anterior side of the mouse egg cylinder, where they form the AVE. Signalling pathway inhibition from the AVE restricts gastrulation towards the opposite side of the AVE, where a combination of WNT, BMP4 and NODAL induces the key mesoderm factor *T* (*Brachyury*)^39,61,70^. Primitive streak formation is initiated at the proximal posterior pole of the epiblast, where cells undergo epithelial-to-mesenchymal transition and differentiate into mesoderm and endoderm^30^ (Fig. 3).

**Fig. 4:**
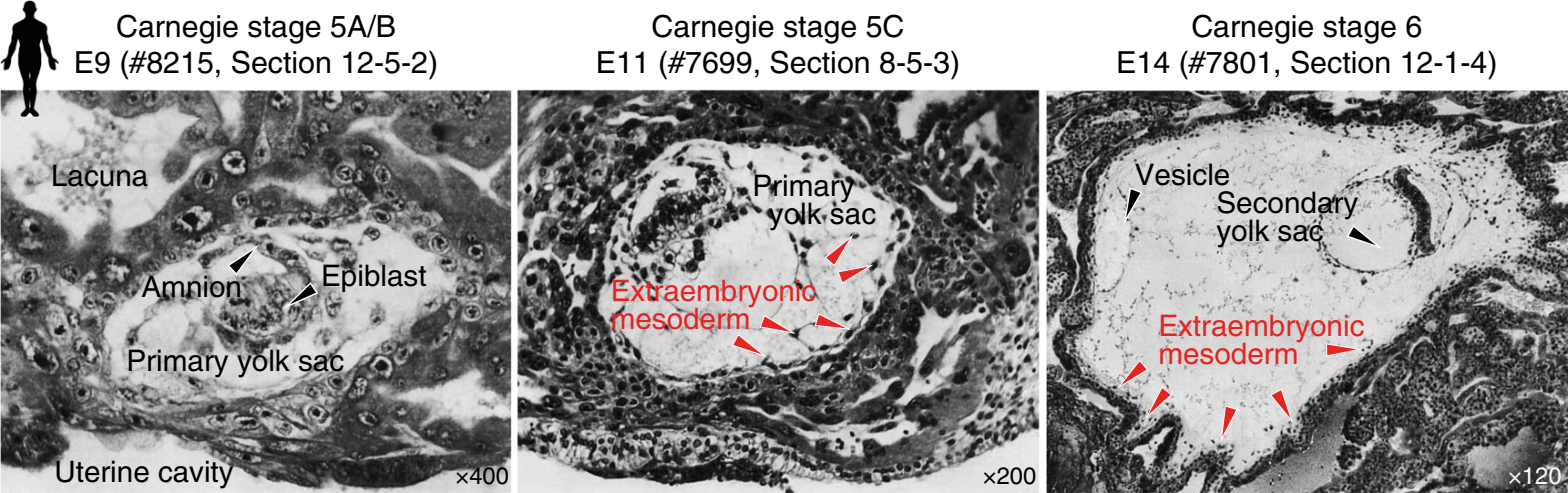
Histology of early implantation stages in human. Embryonic stages are depicted according to Carnegie stage and embryonic day (E) with the Carnegie specimen and section ID indicated. Images are reproduced from ref. ^74^. Original annotations have been removed using the Adobe Photoshop Spot Healing Tool.

Primate AVE formation is associated with local thickening of the visceral endoderm. This can be observed in human embryos as early as CS 5C and 6A^71,72^ (Fig. 4). Equally, the anterior–posterior axis is evident in cynomolgus and rhesus embryos at the same stage (ref. ^73^ and Fig. 12 in ref. ^74^). In cynomolgus, *CER1* is initially expressed throughout visceral endoderm and becomes anteriorly restricted within several hours^73^. The cynomolgus AVE secrets DKK1, presumably to locally inhibit strong WNT3A signalling from the overlying trophoblast and amnion^73^. Prior to gastrulation, BMP4 is expressed in the amnion and subsequently shifts towards the posterior end of the embryonic disc^73^. Moreover, there is robust experimental evidence for a conserved function of FGF, BMP and WNT signalling in pluripotent stem cells (PSCs). Human PSCs correspond to the postimplantation epiblast^34^ and efficiently differentiate into mesoderm and endoderm upon coordinated pathway stimulation mimicking the posterior signalling environment^75,76^. In the mouse, definitive endoderm gradually intercalates with visceral endoderm, although a fraction of extraembryonic cells persists at least until the formation of the early gut tube^77^. The dynamics of visceral endoderm replacement by definitive endoderm in the primate embryo remain unknown and will provide an exciting avenue for further studies.

### The origin of extraembryonic mesoderm in primates

Early extraembryonic mesoderm specification prior to primitive streak formation is a characteristic feature of primate embryogenesis^78,79^ (Fig. 2). This contrasts with rodent development, where extraembryonic mesoderm originates from the first epiblast cells migrating through the primitive streak^61,80^ (Fig. 3). In the human embryo, the early appearance of spindle-shaped cells between the endoderm of the primary yolk sac and trophoblast has been considered mesoderm^71^ or epithelial strands of parietal endoderm origin^74^. This mesh-like array is more pronounced in human and chimpanzee embryos compared to rhesus^81^ and marmoset^82,83^, which may be causally related to the mode of interstitial implantation^74^. In great apes, blastocyst expansion is reduced to allow penetration through the endometrial epithelium, but the parietal endoderm of the primary yolk sac continues to grow during this period at the same rate as in monkeys^74^. It has been suggested that the extensive growth of mesenchymal cells within the blastocyst cavity may play a structural role in the re-expansion of the conceptus after penetration into the maternal endometrium^74^.

The developmental origin of extraembryonic mesoderm is unclear. It initially appears in the periimplantation embryo at CS 4 and thus can theoretically be specified from three possible sources: trophoblast, hypoblast, or epiblast. Originally, extraembryonic mesoderm in early human implantation stages was suggested to be derived from the inner layer of trophoblast, the cytotrophoblast^81,84^. However, subsequent ultrastructural studies have shown that cytotrophoblast and early extraembryonic mesoderm are physically separated by a basal lamina in rhesus^79^ and human^85^. Together with the consistent absence of histological evidence of mesodermal progenitors delaminating from cytotrophoblast in the literature, a cytotrophoblast origin is now regarded as unlikely^79,86^.

Human and non-human primate extraembryonic mesoderm is continuous with, and morphologically indistinguishable from, endodermal cells lining the central cavity of the primary yolk sac^34,87^. Indeed, evidence from rhesus and cynomolgus embryos points towards an origin from the hypoblast lineage^34,79^. Histological and ultrastructural studies by Enders and colleagues show that visceral and parietal endoderm cells of the primary yolk sac delaminate, invade the space between primary yolk sac and cytotrophoblast and differentiate into extraembryonic mesoderm^34,79,86^. Importantly, this hypoblast origin model is supported by electron micrographs of transition stages, where differentiating cells extend towards the underlying basal lamina of the cytotrophoblast, while still attached to parietal endoderm^79^. The invading cells lose apical microvilli and junctional complexes, indicative of epithelial-to-mesenchymal transition. Furthermore, cells in this transition stage show electron-dense bodies within the endoplasmic reticulum cisternae, which constitutes an ultrastructural feature characteristic of extraembryonic mesoderm of later stages^79^. Recent studies support this model, as molecular analysis in cynomolgus embryos revealed in situ expression of endoderm markers GATA4 and GATA6 in early extraembryonic mesoderm cells^34,88,89^. The extraembryonic mesoderm produces large amounts of extracellular matrix, in particular Fibronectin (*FN1*), Collagen (*COL1A1*, *COL1A2*, *COL3A1*, *COL4A1*, *COL6A1* and *COL6A3*) and Laminin111 (*LAMA1*, *LAMB1* and *LAMC1*)^34^. By CS 6, these matrix proteins accumulate around the embryonic stalk region, which connects embryo and placenta^34,79^. Notably, single-cell transcriptome analysis of *GATA4*- and *GATA6*-positive extraembryonic mesoderm cells showed absence of the mesodermal marker *T* (also known as *Brachyury*)^34^. This is in contrast to rodent extraembryonic mesoderm, which is derived from *T*-positive epiblast cells in the proximal region of the primitive streak^80,90,91^. It is tempting to speculate that the epithelial-to-mesenchymal transition in the context of primate extraembryonic mesoderm specification from hypoblast is mediated by a distinct transcriptional circuitry, but functional studies and further embryo profiling data will be required to corroborate or refute this hypothesis.

However, there is a third possible origin of extraembryonic mesoderm. Luckett argues that the posterior margin of the primitive streak of the epiblast develops precociously at CS 5C and early 6, which he considers as the source of all extraembryonic mesoderm^74^. While this hypothesis has been dismissed in the literature^86^ on the grounds that extraembryonic mesoderm is already abundantly present prior to primitive streak formation, it is worth taking a closer look at Luckett’s suggestion (Fig. 4). Indeed, there is histological evidence of an anterior–posterior embryonic axis in human embryos as early as CS 5C. The pronounced thickening of both epiblast and presumptive AVE on one side of the embryonic disc indicates accomplished embryo patterning (Carnegie #8558, #7950 and #8330 in ref. ^71^ and Carnegie #7700 in ref. ^87^). One of these CS 5C specimens even exhibits a pronounced curved shape with precocious delamination of mesoderm at the thinner end of the embryonic disc (#8330,^71^ depicted in Fig. 4). Equally, serial sections of rhesus embryos demonstrate consistent thickening on one side of the embryonic disc in all of the five samples collected at CS 5C^81^. From an evolutionary point of view, an epiblast origin of extraembryonic mesoderm formation would be consistent with rodents and a multitude of other species.

So how can this apparently contradictory evidence be reconciled? We hypothesise that the primary yolk sac serves as a source for extraembryonic mesoderm, ultimately providing an inner mesodermal lining within the former blastocyst cavity (Fig. 2). It is conceivable that the earliest extraembryonic mesoderm originates from hypoblast and is then “supplemented” with mesoderm from the embryonic disc. This hypothesis is consistent with the fact that extraembryonic mesoderm on the abembryonal site (opposite the *embryo proper*) is already abundantly present at CS 5C and 6 (Fig. 4) and thus unlikely to be derived from precocious gastrulation in the embryonic disc. In great apes, primary yolk sac formation might also provide structural support for the re-expansion of the conceptus after interstitial implantation. Once this is achieved, the primary yolk sac collapses into smaller vesicles, which either differentiate or perish in the former blastocyst cavity, now called the exocoelomic cavity. The mesodermal lining of the cytotrophoblast establishes the chorion, which will undergo primary chorionic villus formation in subsequent stages. Deconstruction of the primary yolk sac paves the way for the next chapter of yolk sac development, with the establishment of its successor, the secondary yolk sac.

### Emergence of the secondary yolk sac

Secondary yolk sac formation is a unique feature of primate embryogenesis. Most human specimens at CS 6 exhibit a small secondary yolk sac beneath the embryonic disc, concomitant with multiple vesicles elsewhere in the blastocyst cavity, in particular the abembryonic regions (Fig. 2). This wide variation in structure has led to the concept of primary yolk sac collapse or suggestions that the secondary yolk sac may form by pinching off from the larger primary yolk sac^74^. Alternatively, the swollen spherical nature of the primary yolk sac at CS 5C and 6 might result from turgor. In this case, histological variance could be explained by periodic collapse and re-expansion, a phenomenon commonly observed in the primate blastocyst^92^. The parietal endoderm of the primary yolk sac continues to delaminate extraembryonic mesoderm, while undergoing constant rearrangement resulting in the formation of smaller vesicles. In contrast to the thin and elongated cells of the parietal endoderm and extraembryonic mesoderm, visceral endoderm is densely packed with nuclei, indicating growth and expansion. Therefore, the human secondary yolk sac may be derived from visceral endoderm and potentially smaller fragments of the parietal endoderm of the primary yolk sac^92^.

The concept of secondary yolk sac formation from visceral endoderm is supported from data in New World and Old World monkeys, where blastocysts grow larger compared to great apes and implant superficially. The extensive mesh-like network of extraembryonic mesoderm observed in the primary yolk sac of great apes is reduced to a layer of elongated and spikey parietal endoderm cells in marmoset and cynomolgus^34,82,83^. Visceral endoderm cells at CS 5 and 6 are positive for the proliferation marker Ki67^73^ and form an irregular layer of one to three cells beneath the embryonic disc^34,55^. Detailed molecular characterisation in cynomolgus implantation stages revealed that SOX17-positive visceral endoderm is continuous with the spindle-shaped cells of the primary yolk sac but often folds back at the margins of the embryonic disc (Fig. 2 in ref. ^73^). This leads to the emergence of a small cleft within the visceral endoderm, which sustains specific visceral endoderm marker expression, including SOX17 and FOXA1^34,73^. In contrast, SOX17- and FOXA1-negative parietal endoderm accumulates around the amnion and stalk regions and appears to differentiate into COL6A1-secreting extraembryonic mesoderm^34^. Both extraembryonic mesoderm and visceral endoderm express GATA4 and GATA6, consistent with their common endoderm origin^34^. However, further studies using lineage tracing or spatial transcriptomics in non-human primates will be required to unequivocally clarify the mechanisms of secondary yolk sac formation.

## Functions of the secondary yolk sac in primate embryogenesis

### Haematopoiesis in the yolk sac

**Fig. 5:**
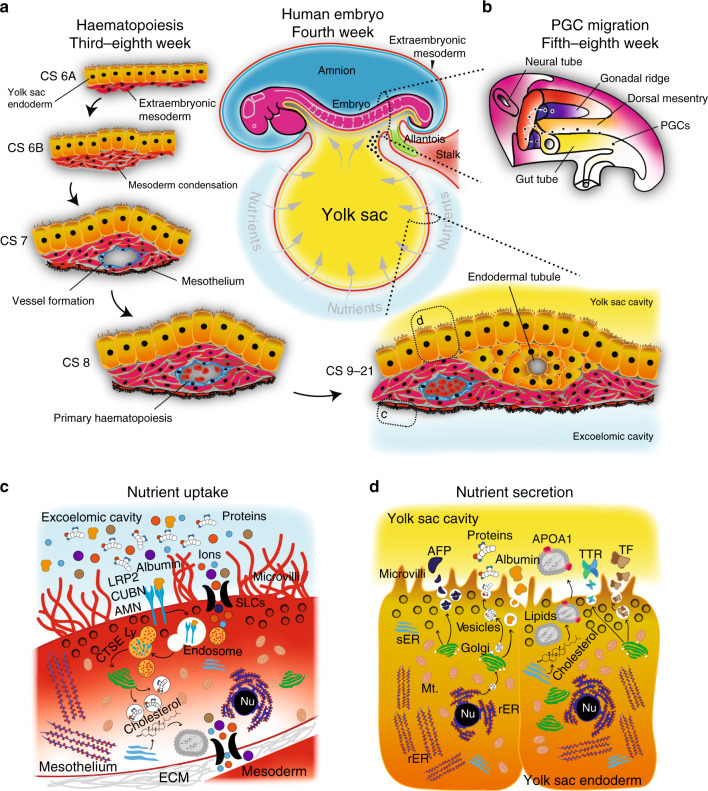
Functions of the primate secondary yolk sac. **a** Vasculogenesis of the yolk sac forms an intricate vascular plexus that envelopes the yolk sac from Carnegie stage (CS) 6A. Haematopoiesis ensues in preparation for the onset of embryonic circulation, mediated by the developing heart. Reciprocal crosstalk signalling between the endoderm and mesoderm regionalises foci of condensed mesoderm, which form primitive blood islands. **b** PGCs are specified in the embryonic region and migrate via the yolk sac into the hindgut and up through the dorsal mesentery towards the genital ridges. Schematic drawn after ref. ^164^. The original images were published in *Medical Physiology* E-Book^164^, Copyright Elsevier (Health Sciences, ISBN 1455733288, 9781455733286). **c** The mesothelium exhibits hallmarks of absorption, degradation and re-synthesis, evident by high concentration of LRP2–CUBN–AMN endovesicular complexes in the plasma membrane. Lysosomes contain hydrolytic enzymes, such as the Cathepsins, to mediate the degradation of maternal proteins and other complex molecules in the nutrient-rich exocoelomic cavity. Nutrients are either transported or re-synthesised and exocytosed either directly into surrounding blood vessels or transported through the extracellular environment towards the endodermal tubules and the yolk sac cavity. **d** The endoderm contains high concentrations of rER, sER, glycogen vesicles and exocytotic vesicles. Yolk sac endoderm cells synthesise and exocytose key carrier proteins such at AFP, TTR, ALB and TF into the yolk sac cavity, which are absorbed by primitive gut endoderm. Nu nucleus, rER rough endoplasmic reticulum, sER smooth endoplasmic reticulum, Mt Mitochondrion, SLC solute carrier family, Ly lysosome, CTSE Cathepsin family member E, LRP2 LDL-related receptor 2, CUBN Cubulin, ApoA apolipoprotein A, TTR transthyretin, TF transferrin, AFP alpha-fetoprotein, AMN amnionless.

Throughout mammalian development, haematopoiesis occurs in three transient waves before haematopoietic stem cells are permanently established in the bone marrow. The first human blood cells are large and nucleated erythrocytes, macrophages and megakaryocytes, which originate in the blood islands of the yolk sac from CS 7 onwards (Fig. 5a)^93–95^.

Blood formation is initiated in yolk sac mesoderm of either embryonic or extraembryonic origin, which aggregates into small masses. These condensations of extraembryonic mesoderm have been postulated to be the primordia of blood islands^96^, which differentiate to form two types of angioblasts: (i) endothelial progenitors, which give rise to constituents of the blood vessels, and (ii) primitive haematopoietic progenitors, encompassing erythroid and myeloid progenitor cells^97–99^. Most blood islands are comprised of haematopoietic cells surrounded by endothelial cells and rapidly develop into an extensive vascular plexus, which envelopes the yolk sac in a sophisticated branching-like pattern. In the mouse, the first primitive erythroid progenitors are considered bipotent, giving rise to unipotent megakaryocytes and erythroid progenitors^100^. The initiation of cardiac contractions marks the onset of the embryo—vitelline circulation, as yolk sac-derived haematopoietic cells are disseminated throughout the developing embryo^101^. Consequently, primate and rodent primitive erythrocytes can be readily found inside the cardiac cavity^102,103^. The first wave of haematopoiesis in the yolk sac is rapidly followed by the second wave, generating erythro-myeloid progenitors and lymphoid progenitors, which transiently seed the foetal liver^104–106^. At the sixth week of human gestation, primitive erythroblasts are found in embryonic and extraembryonic blood vessels, followed by an overall decline of haematopoiesis in the yolk sac from the eighth week^107^. Intraembryonic haematopoietic stem cells arise in the major arteries of the developing embryo through a third and final wave of haematopoiesis^101,108^. Towards the end of gestation, blood cell production has translocated to the bone marrow, which becomes the permanent site for haematopoiesis throughout adulthood in mice and humans^99,101^.

A central question is whether the first blood cells in the primate yolk sac originate from epiblast- or hypoblast-derived extraembryonic mesoderm. Mouse blood island formation appears to be regulated by conserved signalling cascades, including FGF, BMP, TGFβ and WNT pathways, as well as paracrine crosstalk between yolk sac endoderm and the overlying mesoderm^101,108^. Interestingly, mouse yolk sac derived erythro-myeloid progenitors persist into adulthood as a common origin for tissue macrophages^109^. Evidence from human PSCs suggests that mesoderm destined to a haematopoietic fate is marked by primitive streak genes *T* (*Brachyury*), *MIXL1* and *FOXF1*, as well as surface receptors *KDR* and *PDGFRA*^110^. Efficient generation of primitive haematopoietic cells in vitro from nascent mesoderm requires ACTIVIN and BMP signalling^111^ but does not yield long-term haematopoietic stem cells. Conversely, ACTIVIN inhibition^112^ or WNT stimulation^113,114^ of mesoderm progenitors shifts the balance towards definitive haematopoietic lineages, as defined by the capacity to generate T-lymphocytes^112,113^. The fact that primitive and definitive haematopoietic cells can be efficiently derived from human PSCs supports the notion that epiblast-derived mesoderm, at least partially, contributes to yolk sac mesoderm and the first wave of haematopoiesis. Currently, there are no in vitro models for primate hypoblast or hypoblast-derived extraembryonic mesoderm. Further studies and lineage tracing experiments will be required to determine the precise origin of haematopoiesis in primates. While the molecular mechanisms of primary haematopoiesis remain poorly understood, it is clear that the intricate vascular system of the yolk sac plays an indispensable role in transporting nutrients throughout the early stages of gestation. As embryonic development progresses towards organogenesis, the establishment of a complex vascular system is imperative for growth, delivery of nutrients and survival of the conceptus.

### A primordial germ cell vacation in the yolk sac

More than 100 years ago, human primordial germ cells (PGCs) were initially discovered in the yolk sac^115^. PGCs are the founder cells of sperm and egg, which harbour the unique ability to generate a totipotent zygote at fertilisation. The curious fact that PGCs vacate the embryo during the fourth and fifth week of gestation and migrate into the yolk sac shall be the focus of this section.

In rodents, the epiblast acquires uniform competence for the germ cell fate prior to gastrulation; however, the capacity to form germ cells is swiftly confined to the proximal posterior region^116^. Inductive signalling through BMP4 from extraembryonic ectoderm, BMP2 from visceral endoderm and WNT3 from the epiblast are crucial in specifying a cluster of approximately 40 PGCs. The earliest molecular markers include *Prdm1* (Blimp1), *Prdm14* and *Tfap2c* (Ap2γ)^117–119^. Mouse PGCs traverse the primitive streak and allantois towards the hindgut^120^ and undergo genome-wide epigenetic reprogramming, which encompasses X-chromosome reactivation, DNA demethylation and erasure of genomic imprints^121–123^. Their pilgrimage ends in the genital ridges, where germ cells undergo sex-specific maturation in the developing gonads.

PGCs in human and non-human primates were previously considered to originate in the posterior epiblast prior to gastrulation. However, recent evidence in cynomolgus embryos at implantation stages points towards the nascent amnion as a source of germ cells^73^. Primate embryos exhibit a planar, bilaminar structure during the pre-gastrulation period, in contrast to the cup-shaped egg cylinder in rodents. Upon implantation, the primate epiblast segregates amnion and generates the amniotic cavity (Fig. 2). For cynomolgus PGC specification, BMP and WNT signals from the amnion and surrounding cytotrophoblast induce *T* expression in cells of the dorsal amnion, followed by upregulation of *SOX17*, *PRDM1* (*BLIMP1*) and *TFAP2C*^73^. However, the proposed amnion model for PGC formation is not observed in bilaminar embryos of other mammalian species. For instance, porcine PGCs are specified in the posterior embryonic disc through autocrine signals from the epiblast at the early primitive streak stage^124^. Equally, in humans, the epiblast is the likely source of PGCs as suggested by in vitro models of PGC induction from human PSCs^124,125^. Conceivable explanations for a difference between human and Old World monkey germ cell specification have been proposed, including the possibility of human amnion segregation prior to PGC formation or a “dual origin” for PGCs from both amnion and posterior epiblast^126^.

After induction, primate PGCs expand in numbers and enter the yolk sac during gastrulation (Fig. 5). In pre-somite cynomolgus embryos at CS 8, the majority of PGCs reside within the posterior yolk sac at the base of the connecting stalk and the incipient allantois^73^. Importantly, the formation of the primitive gut by lateral folding of the endoderm lining of the yolk sac provides an effective mechanism for PGCs to re-enter the embryo in a central location. Primate PGCs then translocate from within the hindgut into the dorsal mesentery (Fig. 5b) by disrupting the basal membrane of the epithelial lining^127^. Once in the dorsal mesentery, their migration path at the centre of the embryo bifurcates towards the developing gonadal ridges^128,129^. Recent evidence from marmoset embryos has challenged the dogma of active long-range PGC migration, as marmoset PGCs reside in close spatial proximity to the prospective genital ridge^130^. This may suggest “passive translocation” as an important mechanism for primate PGCs to reach their final destination in the developing gonads. At the fifth week of human development, PGCs settle in the genital ridge where subsequent differentiation and patterning of the gonads occurs, dependent upon the karyotype^128,129^.

Collectively, an overarching principle of mammalian germ cell specification emerges, where PGCs are specified at the onset of gastrulation and subsequently moved out of the embryo to escape from somatic signalling bombardment. We hypothesise that the posterior yolk sac provides a respite for nascent PGCs to consolidate the germ cell gene regulatory network and accomplish epigenetic reprogramming in preparation for totipotency. Moreover, the movement of the yolk sac during primitive gut formation serves as an effective means of transportation for PGCs to return back into the embryo and to re-enter in close proximity to the gonadal ridges. The central location of the developing gonads is vital for protection and temperature homoeostasis of mature germ cells in the adult, hence the ability of the yolk sac to deliver PGCs through the hindgut is of fundamental importance. We propose that the yolk sac plays a central role in primate PGC development, acting as both safe harbour and delivery vehicle for the founding cells of the next generation.

### The yolk sac orchestrates histotrophic nutrient transport and metabolism

An efficient and uninterrupted supply of nutrients for the conceptus is a core requirement for successful reproduction. From an evolutionary perspective, the machinery for protein and nutrient transport was already present in the glandular oviduct of our oviparous ancestors, an organ originally responsible for albumin production of the egg. After loss of the calcareous shell, the corpus luteum was recruited to maintain high progesterone levels^131^. This was an essential step to sustain pregnancy and establish histotrophic nutrition through continuous nutrient-rich secretions from the maternal glands^10,131^. Histotrophic nutrition covers the critical time window between fertilisation and the onset of placental function, when a permanent interface between maternal and foetal circulations for gas, nutrient and waste exchange is established in the placental villi. The progression from histotrophic to haemotrophic nutrient uptake is observed in most mammals and marks the end of the first trimester in the human^10,131^.

Histotrophic nutrition is considered the principal nutritional pathway in the first 2 months of human and non-human primate development. The fluid of the exocoelom plays a crucial role in this process by providing a connection between the maternal tissues and the embryo. Although the syncytiotrophoblast of the early placenta efficiently takes up nutrients from uterine secretions and shuttles them into the exocoelomic fluid, it is fundamentally limited with regard to transport capabilities beyond the exocoelomic cavity. Most placental villi are barely vascularised in the first weeks of postimplantation development^71,131^. In contrast, the secondary yolk sac is the first site of haematopoiesis and as such its vasculature is well developed^99^. It is firmly linked to the embryo via the vitelline duct and floats freely in the exocoelomic cavity, bathed in viscous and protein-rich exocoelomic fluid^12,132^. Consequently, the vitelline circulation in the yolk sac provides an effective mechanism for metabolite exchange between the uterus and the developing embryo^15,16,133^.

There are two principal routes for nutrient delivery to the embryo via the yolk sac. The first route is through the blood vessels in the yolk sac wall; the second route is via the yolk sac cavity, which can be regarded as an expansion of the primitive gut. In both cases, metabolites from the exocoelomic fluid are first absorbed by the outer surface layer of mesoderm surrounding the yolk sac, the mesothelium. Metabolites are then shuttled either into the vitelline circulation or the yolk sac cavity. The tissue architecture of the primate yolk sac is especially suited for transport functions. It comprises three layers, an outer mesothelium facing the exocoelomic cavity, a highly vascularised mesenchymal layer in the middle and an inner endodermal epithelium towards the yolk sac cavity^134^. Both the inner endoderm as well as the outer mesothelium display ultrastructural features of an absorptive epithelium, including extensive microvilli, coated pits, glycogen and pinocytotic vesicles^55,135–137^. The absorptive function of endoderm and mesothelium has been demonstrated with peroxidase-uptake assays, which suggest bidirectional nutrient transport in the primate yolk sac^138^. In human, the endoderm expands into a stratified epithelium and forms vessels from the sixth week of gestation (Fig. 5a)^137^. The function of these endodermal tubules remains unknown, but it is tempting to speculate that this network of capillaries further supports the delivery of metabolites.

Nutrient uptake in the yolk sac involves tight regulation of receptor-mediated endocytosis and lysosomal degradation (Fig. 5c), followed by protein synthesis, repackaging and finally exocytosis (Fig. 5d)^55,137,139^. Cathepsins are a small group of protease enzymes found in the low pH environment of lysosomes^140^. The presence of transcripts for Cathepsins (*CTSB, CTSD* and *CTSZ)*^12,141^ indicates conserved proteolytic activity in the chicken, mouse and human yolk sac. Moreover, the human yolk sac epithelium expresses a wide spectrum of solute carrier (SLC) family transporters to shuttle amino acids, glycose, vitamins, nucleoside sugars and ions from the exocoelomic cavity into the yolk sac cavity^12^. Elevated levels of the retinol (vitamin A) transporter transthyretin (TTR) supports the notion of active transport of retinol, which is essential for eye development, retinoic acid synthesis and signalling throughout embryogenesis^142–145^. In addition, mouse and human yolk sac express abundant levels of metal transporters for zinc, iron, folate and vitamins B12, C and E^12^.

Among the most prominent features of the human yolk sac transcriptome are genes associated with cholesterol and lipid metabolism. Cholesterol is required for cell membrane integrity^146^, propagation of signalling pathways^147^ and as a precursor for steroid hormones. Importantly, cholesterol is also essential for the activity of sonic hedgehog proteins^148,149^, which play a focal role in neural development and organogenesis^150^. Shortage or complete absence of cholesterol results in severe birth defects, including holoprosencephaly, heart defects and mental retardation^15,148^. In the human yolk sac, cholesterol uptake occurs via lipoprotein receptors *CUBN* (Cubilin), *LRP2* (Megalin) and *LDLR*^15,151^. After endocytosis, lipids are shuttled into the vitelline circulation via the cholesterol efflux transporter *ABCA1* and lipoprotein complexes containing apolipoproteins^12^. These hydrophobic vesicles are predominantly found in the blood stream, where they effectively deliver cholesteryl esters, triglycerides and essential fatty acids to all parts of the body. In the adult, apolipoproteins are synthesised and secreted by the liver and intestines. However, prior to the development of the foetal liver, the yolk sac epithelium is the primary site of apolipoprotein synthesis (Fig. 5d). The abundant expression of *APOA1*, *APOA4*, *APOB*, *APOE* and *APOC3* in chick, mouse and human yolk sacs points towards an evolutionary conserved function for cholesterol transport and lipid metabolism^12,15^.

Taken together, the primate yolk sac can be considered a pivotal mediator of histotrophic nutrition during the first weeks of embryonic development. It acts as an upgraded extension of the embryonic gut, evolved to take on endocrine and liver-specific functions during organogenesis. The yolk sac ensures efficient nutrient uptake, provides the molecular framework for metabolism and orchestrates the distribution of metabolites to the developing embryo.

## Conclusions and future outlook

**Fig. 6:**
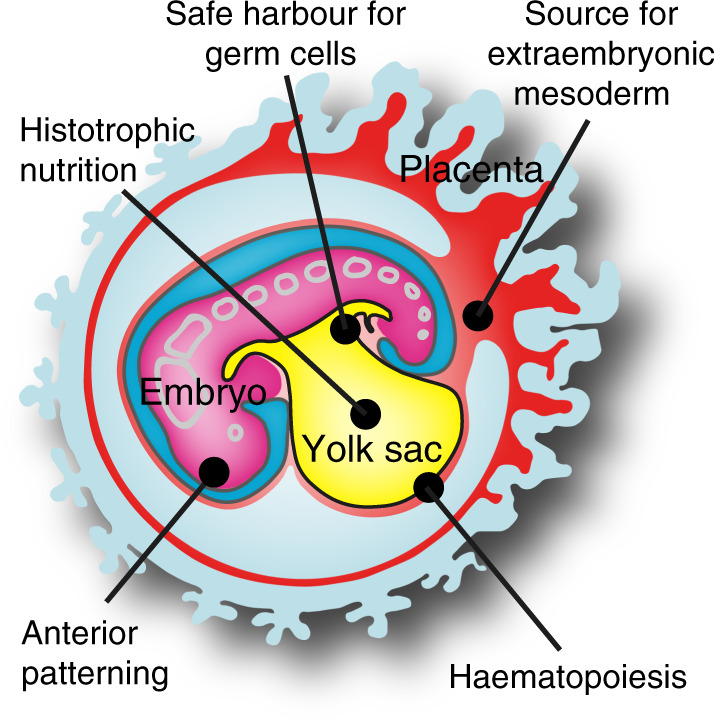
Principal functions of the primate hypoblast and yolk sac. Graphical illustration summarising the roles of the hypoblast—yolk sac lineage during early primate development.

A famous quotation notes that “the embryo is a machine that needs to function while it is being built”^81^. Moreover, embryonic development must ensue in water, as mammalian cell survival strictly depends on an aqueous environment. Extraembryonic membranes accomplish these challenges by providing nutrient transfer, gas exchange and waste management as well as an “aquarium” for the developing conceptus. In primates, the yolk sac constitutes an essential auxiliary device to process and deliver maternal nutrients to the embryo, long before foetal organs and the placenta are sufficiently matured to take on their later functions (Fig. 6). The visceral endoderm has maintained its intricate relationship with the formative cells of the embryonic disc to pattern the anterior–posterior axis. In addition, the hypoblast lineage has adapted to produce extraembryonic mesoderm to cater for the individual modes of primate implantation. The human yolk sac reaches its prime in the first 2 months of gestation, when it gives rise to the first haematopoietic cells and establishes the vitelline circulation, provides shelter for newly specified germ cells and nourishes the rapidly growing embryo (Fig. 6). With the groundwork in place, the yolk sac has fulfilled its function and degenerates. At the end of the first trimester, the human placenta is adequately developed for haemochorial nutrition and the somatic lineages are specified. Organogenesis is now completed and the embryo is ready to enter the foetal growth phase of development.

Many aspects of primate yolk sac formation remain elusive. While embryo profiling in multiple species^23,34,36,50^ revealed an imperative role for WNT signalling in primate hypoblast specification^24^ and the recent generation of naive extraembryonic endoderm from human PSCs provides an exciting avenue^152^, no embryo-derived hypoblast cell lines have been established so far. The quest for the derivation of authentic human and non-human primate hypoblast cells, corresponding transcriptionally and functionally to the in vivo hypoblast, is ongoing and will provide crucial insights into the signalling cascades regulating yolk sac development. Furthermore, hypoblast cell lines will be an effective tool to functionally interrogate the underlying molecular circuitry and constitute the final missing building block to model primate embryogenesis with synthetic embryos. Recent advances in the field of synthetic embryology^153–157^ and protocols to culture human^158,159^ and non-human primate embryos^88,89^ to postimplantation stages may shine light on the enigmatic primary to secondary yolk sac transition, including extraembryonic mesoderm formation. Nevertheless, non-human primate in vivo studies remain imperative to determine transcriptional and epigenetic signatures for thorough assessment of in vitro models. The exciting prospect of extraembryonic organoid cultures^160^ might provide a physiological platform to study yolk sac-specific functions in human, including nutrient uptake, vasculogenesis and haematopoiesis. The latter will be particular revealing if combined with embryonic or extraembryonic mesoderm or both. Ultimately, a deeper knowledge of yolk sac development and its complex functions will be imperative to deconvolute the formation of our most ancient extraembryonic organ.
